# Imaging Features of Primary Intraosseous Adenoid Cystic Carcinoma of the Mandible: A Case Report

**DOI:** 10.7759/cureus.84599

**Published:** 2025-05-22

**Authors:** Sharma Munusamy, Nivashini Ponnampalam, Amali Ahmad

**Affiliations:** 1 Department of Radiology, Kuala Lumpur Hospital, Kuala Lumpur, MYS

**Keywords:** adenoid cystic carcinoma, ct, intraosseous, mandible, mri, perineural invasion

## Abstract

Intraosseous adenoid cystic carcinoma (ACC) of the mandible is an exceedingly rare malignancy. Its subtle onset and aggressive biological behavior, characterized by perineural invasion, present significant diagnostic and therapeutic challenges. We present a case involving a 68-year-old male who experienced persistent pain and swelling in the right hemimandible. Imaging studies using computed tomography (CT) revealed an ill-defined, osteolytic lesion affecting the right hemimandible, leading to cortical destruction. Magnetic resonance imaging (MRI) exhibited an enhancing mass in the right hemimandible, along with evidence of perineural spread along the inferior alveolar nerve. Histopathological analysis confirmed the diagnosis of adenoid cystic carcinoma, displaying a predominant cribriform pattern. The patient subsequently underwent segmental mandibulectomy, followed by adjuvant radiotherapy. This case highlights the essential roles of CT and MRI in the early detection, characterization, and surgical planning for intraosseous ACC. It is crucial for radiologists and clinicians to maintain a high index of suspicion when assessing destructive mandibular lesions, especially those that exhibit perineural spread. Timely diagnosis and a multidisciplinary management approach are vital for optimizing outcomes in this aggressive tumor.

## Introduction

Adenoid cystic carcinoma (ACC) is a malignant neoplasm of the salivary glands, representing around 1% of head and neck malignancies and 10% of salivary gland tumours [1,2]. Intraosseous ACC of the mandible is extremely rare, with only a few cases documented in the literature [3]. Due to its slow-growing and infiltrative nature, as well as its nonspecific clinical presentation, it is often identified at a late stage. Imaging is crucial for accurate diagnosis, surgical planning, and monitoring after treatment.

## Case presentation

**Figure 1:**
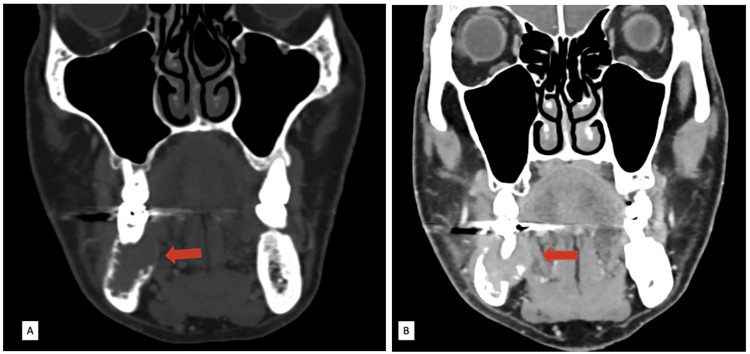
Contrast-enhanced CT neck images in the coronal plane. (A) Bone window setting showing the expanded right hemimandible with cortical erosion (red arrow). (B) Soft tissue window setting showing the soft tissue mass extending to the lingual surface of the gingiva (red arrow).

This case report discusses a 68-year-old male with no significant medical history who presented with electric shock-like pain and numbness in his right jaw. His symptoms had persisted for 10 months. An oral examination revealed a firm swelling at the right posterior alveolar ridge, characterized by buccolingual expansion and mobile molars. Blood investigations, including levels of C-reactive protein and white blood cells, showed no abnormalities. A radiograph indicated an irregular lucent lesion in the right hemimandible. Upon his initial consultation at a private hospital, a contrast-enhanced computed tomography (CT) of the head and neck was performed. This scan revealed an enhancing, expansile mass in the right hemimandible with cortical erosion (Figure 1). This mass extends to both the buccal and lingual surfaces of the gingiva.

**Figure 2:**
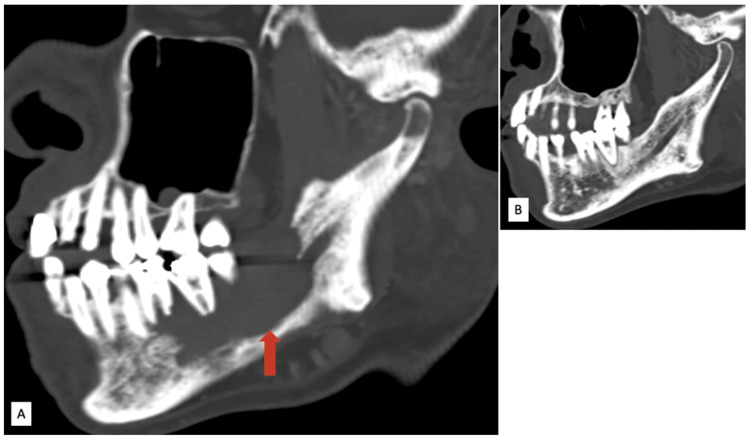
Contrast-enhanced CT neck images in the agittal oblique plane. (A) Lytic lesion in the right hemimandible exposing the right mandibular canal. (B) Normal left hemimandible with an intact mandibular canal for comparison.

**Figure 3:**
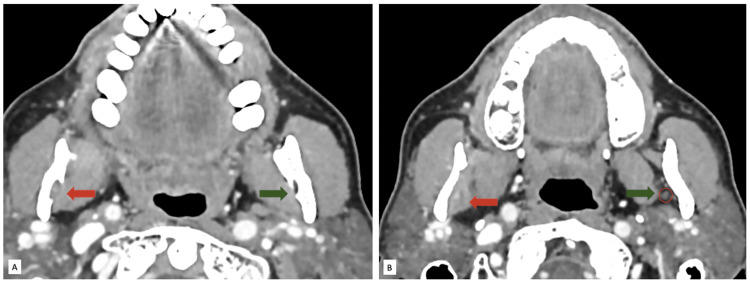
Contrast-enhanced CT neck images in the axial plane at the level of the ramus of the mandible. (A) Widened right mandibular foramen (red arrow); normal mandibular foramen on the left (green arrow). (B) Soft tissue surrounding (red arrow) the right inferior alveolar nerve. Normal inferior alveolar nerve on the left side (red circle) surrounded by fat (green arrow).

The mass exposes the right mandibular canal (Figure 2). Additionally, there was widening of the mandibular canal and thickening of the soft tissue surrounding the right inferior alveolar nerve in the masticator space (Figure 3), suggestive of perineural spread.

**Figure 4:**
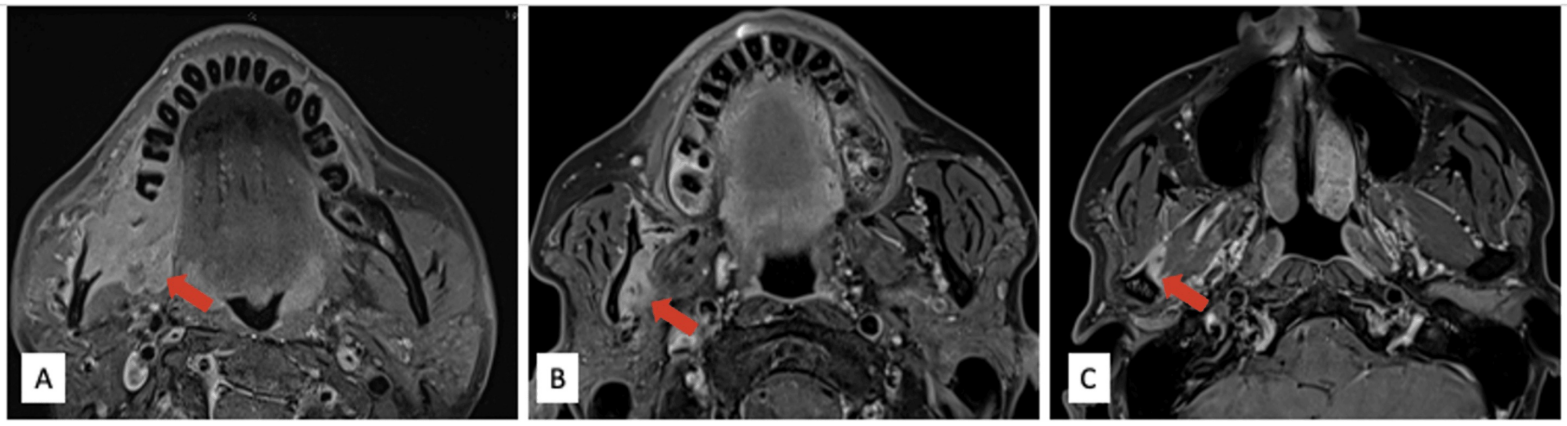
MRI neck axial T1 fat-saturated (T1FS) post gadolinium images. Enhancing mass (red arrow) at the level of (A) the body of the right hemimandible extending to (B) the ramus of the mandible and (C) mandibular condyle with the involvement of adjacent medial and lateral pterygoid muscles.

After the CT scan, the patient was referred to our center for further evaluation. A magnetic resonance imaging (MRI) scan (1.5 Tesla) of the head and neck conducted at our facility demonstrated an enhancing mass at the body of the right hemimandible, which extended to the angle of the mandible and mandibular condyle with involvement of the right medial and lateral pterygoid muscles (Figure 4).

**Figure 5:**
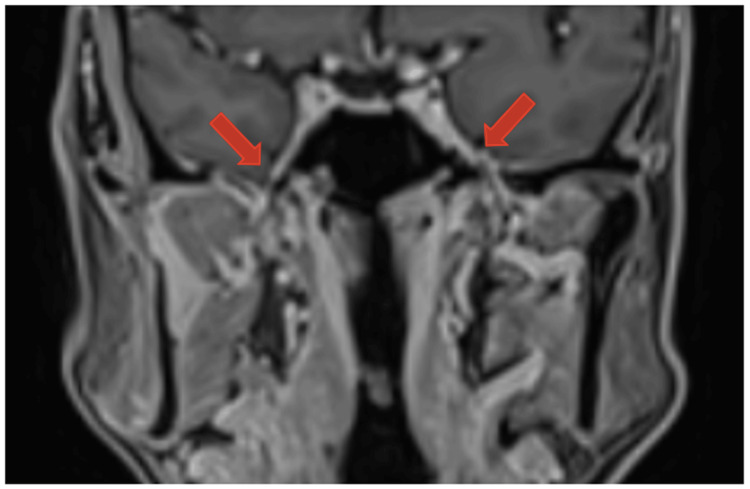
MRI neck coronal T1 fat-saturated (T1FS) post gadolinium image. Bilateral symmetrical appearance of the mandibular division of trigeminal nerves, indicating no intracranial extension of the perineural spread.

The right inferior alveolar nerve appeared thickened, with surrounding soft tissue enhancement indicative of perineural spread. However, the mandibular division of the right trigeminal nerve is preserved (Figure 5).

There were also subcentimeter cervical lymph nodes detected at bilateral level II with preserved fatty hilum. An incisional biopsy of the mass confirmed a diagnosis of adenoid cystic carcinoma. Histological examination revealed the presence of salivary gland cells with infiltration by epithelial and myoepithelial cells, as well as invasions into bone and adjacent fibromuscular tissue organized in solid, tubular, ductal, and cribriform patterns (predominant). The patient underwent a wide local excision of the tumour, including right segmental mandibulectomy, condyle disarticulation, bilateral neck dissection, dental clearance, and reconstruction using a fibula free flap.

Additionally, a dental implant was placed for comprehensive rehabilitation. Following surgery, he underwent radiotherapy, and follow-up CT scans revealed no local recurrence or distant metastasis. The patient has been on regular follow-up and is disease-free for the past two years.

## Discussion

ACC is an uncommon malignancy of salivary gland origin. Intraosseous ACC of the mandible is exceedingly rare, with fewer than 55 well-documented cases reported in the English literature [3-5].

The mandible is a highly unusual site for primary ACC. When it does occur intraosseously, it is hypothesized to arise from ectopic salivary gland tissue trapped during embryologic development or from neoplastic transformation of mucous glands in the lining of odontogenic cysts or sinuses [6,7]. Intraosseous ACCs show a predilection for the posterior mandible, especially the body and ramus, possibly due to the presence of residual salivary gland rests or minor salivary glands in that region.

Patients typically present in the fourth to sixth decades of life, with a slight female predilection [3,8]. Clinical symptoms are often vague and may include pain, swelling, or numbness of the lower lip or chin due to perineural invasion, which is a hallmark of ACC [9].

CT is excellent for assessing osseous involvement and shows a destructive, ill-defined osteolytic lesion. The lesion displays a permeative appearance, often associated with thinning and destruction of cortical plates without significant periosteal reaction [10]. There is no matrix calcification seen. Extension into the mandibular canal or surrounding soft tissue is frequently noted, as seen in our patient.

MRI provides superior contrast resolution and is the preferred modality for evaluating soft tissue extension, marrow involvement, and perineural spread. The lesion usually appears hypo- to isointense on T1-weighted images and hyperintense on T2-weighted images, though T2 heterogeneity may be present due to necrosis or fibrosis [11]. Post-contrast images often demonstrate intense, heterogeneous enhancement with irregular, infiltrative borders [12]. Perineural invasion is best seen on fat-suppressed post-contrast sequences as linear or nodular enhancement along the inferior alveolar nerve [13].

Histopathology is critical in establishing the diagnosis, with ACC demonstrating classic cribriform, tubular, or solid growth patterns.

Due to its infiltrative nature and tendency for perineural and hematogenous spread, wide surgical excision with negative margins is the mainstay of treatment. Postoperative radiotherapy is often indicated, particularly in cases with perineural invasion or close/positive margins [14,15].

Despite aggressive management, ACC is notorious for local recurrence and delayed distant metastasis, most commonly to the lungs and bones [10]. Therefore, long-term follow-up with periodic imaging is essential.

## Conclusions

This case underscores the significance of integrating CT and MRI findings in assessing rare mandibular tumours. Intraosseous ACC should be included in the differential diagnosis of destructive mandibular lesions, particularly when imaging demonstrates infiltrative characteristics and perineural invasion. Therefore, a multimodal imaging approach is crucial for accurate diagnosis, staging, and treatment planning of intraosseous ACC, highlighting its importance in clinical decision-making.
